# Draft genome sequence of *Enterocloster clostridioformis* strain EC-BI isolated from a healthy Jersey calf

**DOI:** 10.1128/mra.00411-26

**Published:** 2026-08-04

**Authors:** Krishna Challa, Kapil Narayan, Nagaraju Indugu, Joe Bender, Sarah Rassler, Alex Post, Terry Webb, Dipti Pitta

**Affiliations:** 1 Department of Clinical Studies, New Bolton Center, School of Veterinary Medicine, University of Pennsylvania 70736, Kennett Square, Pennsylvania, USA; Wellesley College, Wellesley, Massachusetts, USA

**Keywords:** *Enterocloster clostridioformis*, calf fecal, acetogens

## Abstract

We report the draft genome sequence of *Enterocloster clostridioformis* strain EC-BI isolated from the feces of a healthy 2-week-old Jersey calf. The 5.52-Mb genome has a GC content of 49.03%, 98.75% completeness, and encodes 5,152 predicted coding sequences.

## ANNOUNCEMENT

*Enterocloster* species are obligate anaerobic, gram-positive bacteria in the family Lachnospiraceae (1) and important constituents of the mammalian gut microbiota. They ferment carbohydrates, particularly plant-derived polysaccharides, producing short-chain fatty acids (2, 3) that contribute to host energy metabolism. *Enterocloster clostridioformis* has been associated with gut metabolic functions in humans and animals (1, 4), but its presence in the early-life microbiome of ruminants has not been reported. Here, we report the draft genome sequence of *E. clostridioformis* strain EC-BI isolated from a healthy 2-week-old Jersey calf.

Fecal samples were collected from healthy calves (1–2 weeks old) at a dairy farm in Pennsylvania, USA. Samples were cultured anaerobically in pre-reduced yeast casitone fatty acids (YCFA) medium at 37°C for 24–48 h. Serial dilutions were prepared using YCFA agar roll tubes to obtain pure colonies. Colonies were subcultured seven to eight times to obtain pure cultures, and purity was verified by Gram staining and denaturing gradient gel electrophoresis, and cell morphology was examined by scanning electron microscopy.

Genomic DNA was extracted using the repeated bead beating plus column (RBB + C) method (5); the same DNA preparation was used for both 16S ribosomal RNA (rRNA) gene sequencing and whole-genome sequencing. DNA quality and concentration were assessed using a spectrophotometer, yielding a DNA concentration of 456.2 ng/µL and an A260/A280 ratio of 2.02, indicating high-quality DNA. The V1–V2 region of the 16S rRNA gene was amplified using primers F27 (5′-AGAGTTTGATCCTGGCTCAG-3′) and R338 (5′-TGCTGCCTCCCGTAGGAGT-3′) (6) and Sanger sequenced, showing 99.45% sequence identity to *Enterocloster clostridioformis* strain NR_044715.2.

Whole-genome sequencing libraries were prepared using the Nextera Flex DNA Library Preparation Kit (Illumina, San Diego, CA, USA) according to the manufacturer’s instructions, with no protocol modifications, and sequenced on an Illumina HiSeq platform (2 × 150 bp). Reads were processed using fastp v1.0.1 (7) for adapter trimming and quality filtering, including removal of low-quality bases (Phred score < 20), ambiguous bases, and reads < 50 bp. Duplicate reads were removed using clumpify v39.85 (8), and host contamination was filtered using Bowtie2 v2.3.4.3 (9) against human and bovine reference genomes. A total of 5,470,329 high-quality reads were assembled *de novo* using SPAdes v3.15.5 (10) and polished with Pilon v1.24 (11). Assembly statistics were generated with QUAST v5.3.0 (12), genome quality was assessed using CheckM v1.2.3 (13), taxonomic classification was performed with GTDB-Tk (14), annotation was completed using the National Center for Biotechnology Information Prokaryotic Genome Annotation Pipeline (PGAP) v6.10 (15), and antimicrobial resistance genes were identified using AMRFinderPlus v4.0.23 (16). Default parameters were used unless otherwise noted.

The draft genome consisted of 185 scaffolds totaling 5,523,039 bp, with an N50 of 78,619 bp and a GC content of 49.03% (Table 1). CheckM estimated 98.75% completeness and 3.21% contamination, with ~300× genome coverage. PGAP predicted 5,504 genes, including 5,152 protein-coding genes, 279 pseudogenes, 5 rRNA genes, 64 transfer RNA (tRNA) genes, 1 RNase_P_RNA gene, 1 non-coding RNA (ncRNA) gene, and 2 other genes. AMRFinderPlus identified putative resistance genes *aad9*, *ant (6)-Ia*, *aph(3′)-IIIa*, *sat4*, *tet(40)*, *tet(O)*, and *vanR*.

**TABLE 1:** Genome assembly, quality, classification, and annotation statistics

Category	Metric	Statistic
Assembly statistics	Number of contigs	188
	Number of scaffolds	185
	Largest contig (bp)	227,428
	Total length (bp)	5,523,039
	GC (%)	49.03
	N50 (bp)	78,619
CheckM quality metrics	Completeness (%)	98.75
	Contamination (%)	3.21
GTDB-Tk classification	Closest genome classification	*Enterocloster clostridioformis*
	Closest reference genome	GCF_020297485.1
	Average nucleotide identity (%)	99.7
PGAP annotation	Total genes	5,504
	Protein-coding	5,152
	Pseudogenes	279
	rRNA genes	5
	tRNA genes	64
	RNase_P_RNA	1
	ncRNA	1
	Other genes	2
AMR profiles	Putative AMR genes	*aad9*; *tet(40)*; *ant(6)-Ia*; *sat4*; *aph(3′)-IIIa*; *tet(O)*; *vanR*

## Data Availability

This Whole Genome Shotgun project has been deposited in GenBank under the accession number JBWJAI000000000. The raw sequencing reads have been deposited in the Sequence Read Archive under accession number SRR37736293. The partial Sanger-derived 16S rRNA gene sequence is available in GenBank under accession number PZ596596.
